# An overview of molecular markers for identification of non-human fecal pollution sources

**DOI:** 10.3389/fmicb.2023.1256174

**Published:** 2023-11-23

**Authors:** Tanja Zlender, Maja Rupnik

**Affiliations:** ^1^National Laboratory of Health, Environment and Food, Centre for Medical Microbiology, Maribor, Slovenia; ^2^Department of Microbiology, Faculty of Medicine, University of Maribor, Maribor, Slovenia; ^3^Biotechnical Faculty, University of Ljubljana, Ljubljana, Slovenia

**Keywords:** fecal source tracking, microbial source tracking, fecal pollution, host-specific markers, animals

## Abstract

Identifying primary sources of fecal pollution is important for assessing public health risks and implementing effective remediation strategies. To date, one of the main molecular approaches for identifying sources of fecal pollution relies on detecting molecular markers within bacterial, viral, or mitochondrial nucleic acids, that are indicative of a particular host. With a primary focus on identifying fecal pollution originating from humans, the field of fecal source tracking often places less emphasis on livestock sources, frequently leaving the problem of wildlife fecal pollution unaddressed. In this review, we summarize 55 previously published and validated molecular assays and describe methods for the detection of molecular markers that are indicative of non-human hosts. They cover a range of 15 animal species/groups with a primary focus on domestic animals including cattle, pigs, dogs, and poultry. Among assays associated with wild animals, the majority are designed to detect bird feces, while the availability of assays for detecting feces of other wild animals is limited. Both domestic and wild animals can represent a zoonotic reservoir of human enteropathogens, emphasizing the importance of their role in public health. This review highlights the need to address the complexity of fecal contamination and to include a broader range of animal species into assay validation and marker identification.

## 1 Introduction

Fecal pollution of surface and groundwater poses a major risk to human, animal, and environmental health (Paerl et al., 2003; Soller et al., 2010; Penakalapati et al., 2017). Identifying primary sources of fecal pollution is important for two main reasons: for assessing public health risks (Ashbolt et al., 2010; Soller et al., 2010) and implementing effective remediation strategies (Tran et al., 2015).

The level of public health risk can vary depending on the origin of fecal contamination, as different hosts can harbor varying types and amounts of pathogens (Fong and Lipp, 2005; Soller et al., 2010, 2014; Penakalapati et al., 2017). Human-derived fecal pollution is predominantly associated with exposure to human enteric viruses, characterized by low infectious doses (Haas et al., 1993; Fong and Lipp, 2005). While the transmission of animal enteric viruses to humans is generally limited (Delahoy et al., 2018), other pathogens found in animal feces, such as bacteria (*Campylobacter* spp., non-typhoidal *Salmonella*, shiga-toxin producing *Escherichia coli*), and protozoans (*Giardia duodenalis*, *Entamoeba histolytica*, *Toxoplasma gondii*, *Cryptosporidium* spp.) can pose a significant threat (Kotloff et al., 2013; Torgerson and Mastroiacovo, 2013; Daniels et al., 2015; Penakalapati et al., 2017; Delahoy et al., 2018).

The need for identifying primary sources of fecal contamination has driven extensive research in this field for over two decades (Bernhard and Field, 2000a,b; Scott et al., 2002). A set of methods and techniques used to identify the origin of fecal pollution is called fecal source tracking (FST) and methods that target microbial nucleic acids exclusively are referred to as microbial source tracking (MST) methods (Scott et al., 2002; Simpson et al., 2002; Stoeckel and Harwood, 2007; Boehm et al., 2013; Stewart et al., 2013; Steinbacher et al., 2021). Although less common, other potential targets for FST include mitochondrial DNA (mtDNA) of target hosts (Martellini et al., 2005; Caldwell et al., 2007; Schill and Mathes, 2008; Caldwell and Levine, 2009; He et al., 2016). Additionally, a non-marker-based chemical approach can be used to identify sources of fecal pollution due to distinct chemical compositions present in the feces of different animals. This includes the detection of fecal sterols, fatty acids, pharmaceuticals, caffeine and chemical sweetners (Scott et al., 2002; Lu et al., 2016; Staley et al., 2016).

While FST research primarily emphasizes the detection of human fecal contamination, it is crucial to acknowledge that feces from wild and domestic animals can significantly contribute to fecal pollution of recreational and drinking water and serve as a significant source of pathogen exposure for both humans and animals (Ashbolt et al., 2010; Soller et al., 2010; Daniels et al., 2015; Penakalapati et al., 2017; Rashid et al., 2019). A variety of assays for the detection of non-human fecal contamination have been developed and tested for their diagnostic (e.g., sensitivity, specificity, accuracy), analytical (limit of detection, limit of quantification) and biological (persistence, resistance, mobility) performance criteria. However, most existing assays require further validation, particularly concerning their key biological attributes. While the need for validation of existing markers remains, there is still a potential for exploration of undiscovered wild and domestic animal-associated markers that may be facilitated by the utilization of next generation sequencing (Ohad et al., 2016; Boukerb et al., 2021).

## 2 Literature search

To find publications that evaluated the performance of FST assays identifying fecal contamination of animal origin literature search was performed using the following bibliographic databases: PubMed, ScienceDirect, ResearchGate and Google Scholar. Keywords used for the search included: microbial source tracking OR fecal pollution source tracking OR host-specific markers OR animal-specific markers. These keywords were searched in combination (AND) with performance OR validation and in some cases also with fecal contamination OR fecal pollution OR animal feces. The search was limited to the English language and included papers published before October 2022.

We obtained 70 publications for article review, out of which 46 matched our criteria. The publication selection criteria were: (1) article is peer-reviewed, (2) includes assays for identifying non-human fecal pollution sources, (3) contains information about experimentally determined assay specificity and/or sensitivity using end-point PCR, real-time PCR, digital PCR or isothermal amplification methods, (4) the assay specificity is given as a single number for each assay and (5) the evaluated assays target bacteria, viruses, or host mtDNA.

## 3 Target genes of fecal source tracking assays

The host-associated nature of gut microbiota makes microbial genes suitable markers of the fecal pollution source. One of the most targeted genes in marker-based FST techniques is the bacterial 16S rRNA gene (Harwood et al., 2018). The 16S rRNA gene is well conserved among bacteria of the same species but also contains variable regions providing a tool for distinguishing microbial species and differentiating between different hosts (Zhang et al., 2012). In addition, bacterial cells generally have multiple copies of nearly identical 16S rDNA (Acinas et al., 2004), which increases the sensitivity of its detection (Zheng and Shen, 2018). Other genes associated with a specific host can be involved in host–microbe interactions, pathogenesis or have other, sometimes unknown functions (Khatib et al., 2002; Hamilton et al., 2006; Shanks et al., 2006, 2008; Ufnar et al., 2007; Schill and Mathes, 2008; Yampara-Iquise et al., 2008; Aslan and Rose, 2013; Zhuang et al., 2017; Somnark et al., 2018a).

Bacteria from the order *Bacteroidales* are the most targeted taxon in FST (Bernhard and Field, 2000a; Boehm et al., 2013; Harwood et al., 2014; Ahmed et al., 2016b; Ahmed and Harwood, 2017). These bacteria are anaerobic and limited to feces, animal rumen, and other cavities of humans and most warm-blooded animals (Paster et al., 1994). In the gut, they outnumber fecal coliform bacteria by two to three orders of magnitude (Meays et al., 2004; Zhang et al., 2012). In bird intestines their abundance is substantially lower (Lu et al., 2007, 2008). Therefore, assays targeting birds include bacteria from genera *Lactobacillus* (Ohad et al., 2016; Vadde et al., 2019; Rytkönen et al., 2021), *Helicobacter* (Green et al., 2012; Vadde et al., 2019), *Brevibacterium* (Weidhaas et al., 2010; Weidhaas and Lipscomb, 2013; Schiaffino et al., 2020) and *Catelicoccus* (Lu et al., 2008; Kirs et al., 2011).

Due to high stability of viruses in the environment and their host specificity, viral markers are suitable candidates for tracking sources of fecal contamination of human and non-human origin (Noble and Fuhrman, 2001; Ley et al., 2002; Ahmed and Harwood, 2017). Host-associated markers can be found in teschoviruses (Jiménez-Clavero et al., 2003), adenoviruses (de Motes et al., 2004; Hundesa et al., 2006; Ahmed et al., 2010), polyomaviruses (Hundesa et al., 2006) and enteroviruses (Ley et al., 2002). Having similar characteristics to enteric viruses, bacteriophages have also been proposed as promising tools for detection of fecal pollution (Toribio-Avedillo et al., 2021).

Host mtDNA (human or non-human) can also be used as a marker based on the presumption that it is highly abundant in feces (Caldwell et al., 2007). Mitochondria are found in all cells of eukaryotes and each mitochondrion contains multiple copies of its own genome. Therefore, false positive results can be obtained by detecting non-fecal sources (i.e., skin cells) (Caldwell and Levine, 2009).

## 4 Molecular techniques for identifying sources of fecal contamination

Various PCR technologies are used for identification and/or quantification of host-associated markers, however, isothermal nucleic acid amplification techniques such as loop-mediated isothermal amplification (LAMP) and helicase-dependent amplification (HDA) can be utilized. The PCR technologies used for identifying primary sources of fecal pollution include end-point PCR, real-time PCR, and digital PCR (dPCR).

### 4.1 End-point PCR

End-point PCR is a cost-effective method for amplification of FST markers. The resulting amplicons are typically visualized on an agarose gel containing an intercalating dye that emits fluorescence under UV light (Fremaux et al., 2009; Shanks et al., 2010; He et al., 2016; Somnark et al., 2018a; Ballesté et al., 2020). Alternatively, there are more rapid, sensitive, and automated visualization techniques available, including microchip electrophoresis and microfluidics-based microchip platforms (Chen et al., 2022; Zeid et al., 2023).

### 4.2 Real-time PCR

Due to end-point PCR having multiple limitations, including lack of quantification, sensitivity-specificity trade-off and post-PCR processing, most FST assays were later adapted from end-point to real-time PCR, which is less labor-intensive and offers a rapid detection of markers with higher sensitivity, specificity, and accuracy. Another major improvement from end-point PCR is that real-time PCR can be used for quantification. Quantitative real-time PCR (qPCR) is based on real-time detection of fluorescence signals that are emitted either by hybridization of the PCR product to a sequence-specific probe labeled with a fluorescent reporter or by binding of the intercalating dye into double stranded PCR products. In FST TaqMan probes and SYBR green dye are most frequently used among probe and dye-based qPCR chemistries respectively (Cao and Shockey, 2012; Shahraki et al., 2019; Schiaffino et al., 2020). TaqMan assays tend to be more specific and are considered a better choice for detecting host-associated markers in environmental samples. When using SYBR green, melting curve analysis can be used to tackle specificity issues, but this may compromise accurate quantification (Kildare et al., 2007).

Considering disadvantages, qPCR has been linked to quantification errors due to PCR inhibition (Noble et al., 2010; Green and Field, 2012; Cao et al., 2013) and low reproducibility due to usage of standard reference materials from different vendors or batches (Sivaganesan et al., 2011; Cao et al., 2013). In an effort to minimize these limitations, USEPA and NIST developed a Standard Reference Material 2,917 (NIST SRM® 2,917) that functions with 13 recreational water quality qPCR assays including Rum-2-Bac, CowM2, CowM3, DG3, DG37, Pig-2-Bac and GFD among non-human markers of fecal pollution (Willis et al., 2022).

### 4.3 Digital PCR

Another variation of PCR – dPCR has emerged as a promising and reliable tool for the detection of molecular markers. It works by partitioning the sample into many individual reactions, each containing a target molecule or no target at all. Each microreaction undergoes PCR amplification separately and microreactions with and without amplified product are individually counted. Therefore, this method provides absolute quantification without relying on external references and curves (Rački et al., 2014; Taylor et al., 2015; Devonshire et al., 2016). However, it comes at a higher cost and has a relatively narrow dynamic range due to the saturation of positive reactions in high target concentrations (Tang et al., 2016; Zhao et al., 2016). Both dPCR and qPCR assays can be multiplexed to simultaneously detect multiple FST targets (Caldwell et al., 2007; Wolf et al., 2010; Ishii et al., 2014; Staley et al., 2018).

### 4.4 Isothermal amplification methods

LAMP is an alternative to PCR that operates at a constant temperature (usually 60-65°C) and enables us to detect markers rapidly and at a low cost. It can be used on-site without the DNA extraction step and in facilities with limited resources. LAMP is performed using a set of two or three primer pairs and involves a DNA polymerase with a high strand-displacement activity (Nagamine et al., 2001; Martzy et al., 2017; Wang et al., 2023). The amplified products can be detected using various methods, including agarose gel electrophoresis (Nagamine et al., 2001), turbidimetry (Mori et al., 2001; Huang et al., 2017), technologies employing fluorescence and colorimetric detection (Jiang et al., 2018; Wang et al., 2023). Francois et al. (2011) found that the DNA polymerase used in LAMP (*Bst*) is less susceptible to inhibitory substances present in stool, urine, and blood in comparison with other polymerases such as *Taq*. Additionally, LAMP can be quantitative and used to detect multiple targets simultaneously using a LAMP-based microfluidic chip (Jin et al., 2021). Considering disadvantages, this method requires complex primer design (Gadkar et al., 2018) and can often lead to the detection of false positive results (Kuboki et al., 2003; Abbasi et al., 2016; Gadkar et al., 2018). The problem of detecting false positives can be reduced by replacing intercalating dyes with labeled oligonucleotide strand displacement (OSD) probes, that work similarly to TaqMan probes in PCR (Jiang et al., 2015).

In the field of FST, a general *Bacteroidales* PCR (Wang et al., 2023) and a human-associated (HF183) assay have been successfully adapted for LAMP (Jiang et al., 2018). However, there are currently no LAMP-compatible assays designed to detect fecal pollution deriving from non-human animals.

An additional approach can be used for the detection of FST markers without relying on trained personnel and specialized equipment. It utilizes HDA along with a lateral-flow strip test and requires only a heating block for amplification. The major limitation of this method lies in its inability to quantify results (Kolm et al., 2019).

## 5 Evaluation of fecal source tracking assay performance

Evaluation of FST assay performance requires a comprehensive assessment of various crucial aspects, including diagnostic, technical, analytical, and biological.

### 5.1 Diagnostic measures and marker abundance

The most common diagnostic measures to facilitate the selection of the best FST assay are specificity and sensitivity. Specificity refers to the proportion of samples that are not the target of interest and correctly yield a negative result, whereas sensitivity represents the proportion of target samples in which the marker is detected (EPA, 2005; Kildare et al., 2007). Positive predictive value, negative predictive value, and accuracy can complement the specificity and sensitivity calculations (Kildare et al., 2007). It has been suggested that there is no universally acknowledged performance benchmark that categorizes an assay as appropriate for FST; instead, the selection criteria are case-dependent (Belanche and Blanch, 2011; Harwood and Stoeckel, 2011; Raith et al., 2013; Reischer et al., 2013; Demeter et al., 2023). Caution should be taken when interpreting diagnostic measures, as they are heavily reliant on sampling intensity, the choice of animal species to be sampled and sampling location.

Due to geographical instability, diagnostic measures of a marker should always be validated before its application in a new geographical area (Bernhard and Field, 2000a; Scott et al., 2005; McQuaig et al., 2006; Ufnar et al., 2006; Stoeckel and Harwood, 2007; Harwood et al., 2009). Furthermore, potential temporal instability of markers is another important factor to consider (Reischer et al., 2013; Yahya et al., 2017; Mayer et al., 2018; Ballesté et al., 2020).

Another critical consideration revolves around marker abundance. Highly abundant markers are more likely to be detected, particularly in situations where microbial contamination is present in low concentrations (Roslev and Bukh, 2011). Units of measure for abundance are typically expressed as number of gene copies per unit of fecal wet mass on a logarithmic scale (Raith et al., 2013; Yahya et al., 2017; Vadde et al., 2019; Schiaffino et al., 2020; Zhang et al., 2020).

Ideally, a marker would exhibit 100% specificity and 100% sensitivity for a given target host. However, this level of performance is frequently not achieved, as shown in Supplementary Table 2. One approach to overcome problems of low sensitivity, specificity, marker abundance and quantification abilities is to use multiple markers of fecal pollution for detection of one animal group (Ballesté et al., 2010; Ahmed et al., 2019; Liang et al., 2020).

### 5.2 Technical and analytical measures

The MIQE guidelines (Minimum Information for Publication of Quantitative Real-Time PCR Experiments), published by Bustin et al. (2009), provide a standardized framework for generating consistent and high-quality real-time PCR data. Additionally, guidelines for digital PCR were published in 2013 and subsequently updated in 2020 (Huggett et al., 2013; Whale et al., 2020).

Among important analytical criteria for establishing marker detection and quantification thresholds are assay limit of detection (aLOD) and assay limit of quantification (aLOQ). Both parameters indicate how effectively an analytical method can detect (aLOD) or quantify (aLOQ) a specific marker under ideal laboratory conditions. In theory, PCR assays can identify very low numbers of gene copies (Santo Domingo et al., 2007; Armbruster and Pry, 2008; Demeter et al., 2023). However, in practice, the analytical sensitivity is influenced by the characteristics of the sample matrix and the steps involved in sample processing (Demeter et al., 2023).

While aLOD and aLOQ focus on the method’s theoretical capability, sample limit of detection (sLOD) and sample limit of quantification (sLOQ) account for the actual sample matrix in which the analysis is conducted and consider the effect of all sample processing steps including sampling, filtration, nucleic acid isolation and the amount of nucleic acid analyzed (Santo Domingo et al., 2007; Kolm et al., 2019; Demeter et al., 2023). These parameters help assess whether the FST method is appropriate for detecting the marker in specific types of environmental samples considering the chosen methodology (Reischer et al., 2006; Weidhaas et al., 2010; Green et al., 2012; Devane et al., 2013; Kolm et al., 2019).

aLOD, aLOQ, sLOD, and sLOQ should be expressed with a certain level of confidence (usually 95%) (Reischer et al., 2006; Armbruster and Pry, 2008; Bustin et al., 2009; Devane et al., 2013; Kolm et al., 2019; Schiaffino et al., 2020), however this detail is often not stated in the research papers. In the context of FST, aLOD and aLOQ values are typically expressed as the number of gene copies per reaction, whereas the reported units of measure for sLOD and sLOQ lack consistency (see Supplementary Table 2).

To account for the impact of the water sample matrix on the detection of host-associated markers, the process of establishing sLOD and sLOQ involves diluting feces of a target host in environmental water samples (freshwater, estuarine and marine water). In some studies, sLOD and/or sLOQ were determined by diluting fecal samples in distilled water or buffers, accounting only for the fecal sample matrix (Supplementary Table 2).

In validation studies, the information about analytical and sample limits of detection and quantification is sometimes not stated, as indicated by the data presented in Supplementary Table 2. Nonetheless, these limits provide a crucial means to assess method sensitivity and potential bias, especially when dealing with extremely low marker concentrations. Additionally, they significantly impact the interpretation of diagnostic data (Raith et al., 2013).

### 5.3 Biological measures

Key biological attributes of FST markers include persistence, resistance, and mobility. Persistence refers to the viability of the indicator organism or molecular detectability of the marker in a water sample. It is known to be influenced by abiotic and biotic factors such as sunlight, temperature, and salinity (Haugland et al., 2005; Siefring et al., 2008; Demeter et al., 2023). Ideally, the molecular detectability of the marker would match the decay rates of waterborne pathogens (Harwood et al., 2014; Demeter et al., 2023).

Technical treatment, and chemical substances such as disinfectants, antibiotics and metals can also affect viability and molecular detectability of the marker. Understanding which factors affect indicator organism and marker concentrations will help us understand their resistance in natural environment (e.g., in wastewater treatment systems) (Steinbacher et al., 2021; Demeter et al., 2023).

In environmental waters, different indicators (e.g., bacterial, viral) have different movement patterns, which can affect FST measurements. Apart from having different sedimentation rates, some indicators tend to attach to particles while others disperse freely in the water. In flowing watercourses, the latter tend to be transported more rapidly (Devane et al., 2022; Wang et al., 2022; Demeter et al., 2023).

While numerous genetic markers have been identified, our understanding of their key biological attributes remains very limited. These attributes, including persistence, resistance, and mobility, are vital factors in assessing the performance of host-associated assays in different water environments, considering both biotic and abiotic factors (Demeter et al., 2023).

### 5.4 Assessing true positive probability in environmental marker detection

When a marker is tested on water samples, we can calculate conditional probability that the detection of a host-associated marker in a given water sample is the result of true positivity using Bayes’ theorem. To perform this calculation, additional information regarding the probability of fecal contamination from a specific host is required for each tested water body (Kildare et al., 2007). If the given probability is unknown, the result can be calculated by varying the prior probabilities from 0 to 1 as described by Lamendella et al. (2009).

## 6 Assays and validations for tracking animal fecal pollution

**Figure 1:**
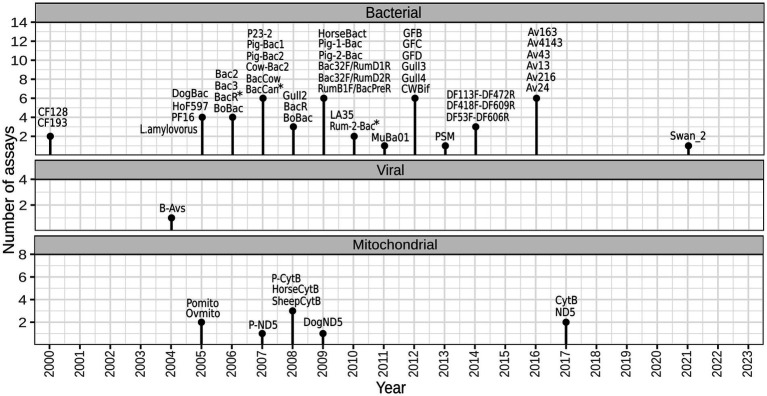
Temporal diagram representing the development of animal-associated markers for identifying sources of fecal pollution. The figure was created with R 4.2.2 using package ggplot2. *forward and/or reverse primer were modified in one or more validation studies.

A total of 55 assays have been published for tracking fecal pollution of animal origin, mostly between years 2005 and 2017 (shown in Figure 1, listed in Supplementary Table 1). They are designed to target a range of diverse genetic markers located within different genes, including the bacterial 16S rRNA gene, viral hexon gene, and host mtDNA. Certain publications have made slight modifications to some of these assays in terms of primer sequences (Yahya et al., 2017; Kolm et al., 2019; Rytkönen et al., 2021; Yasar et al., 2021).

**Figure 2:**
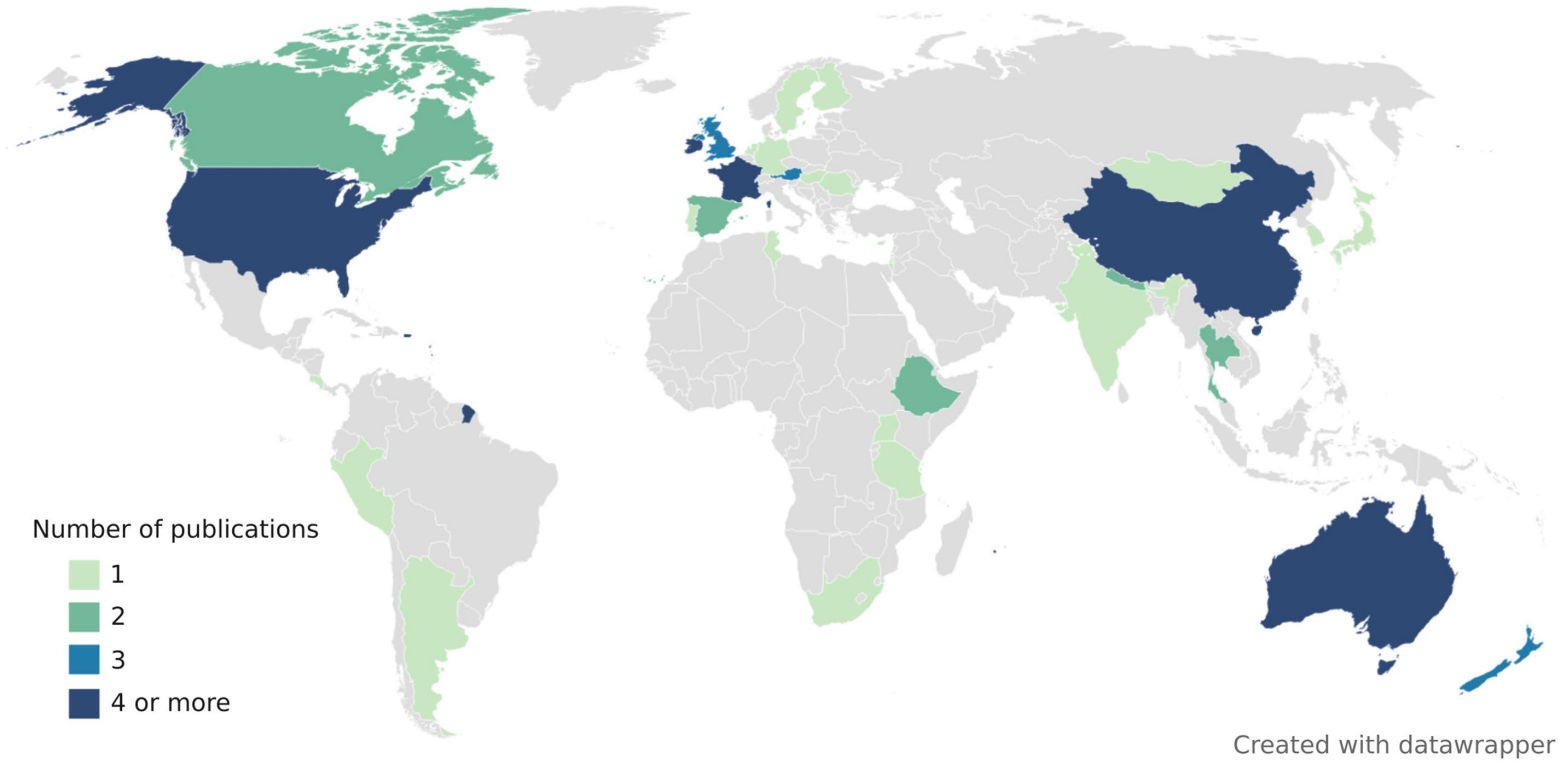
World map representing the number of publications evaluating the performance of animal-associated markers of fecal contamination. Some publications conducted validation studies over multiple countries. All the publications included in this map were peer-reviewed, contained information about assay specificity and/or sensitivity and included assays that target either bacteria, viruses, or host mtDNA.

The choice of FST assays is difficult as host-associated markers may be geographically and temporarily unstable (Reischer et al., 2013; Yahya et al., 2017; Mayer et al., 2018). Ideally, each laboratory should find an appropriate assay in their geographical area by conducting their own validation studies. Alternatively, markers previously validated in their geographical area may be used. FST studies have been conducted all around the world, but mainly in United States, China, Australia, New Zealand, and Europe (France, Ireland, Austria, and the United Kingdom) (Figure 2). Overview of publications, assays (primer and probe sequences, amplicon lengths, annealing temperatures, validation metrics) and tested samples can be found in Supplementary Table 2.

**Figure 3:**
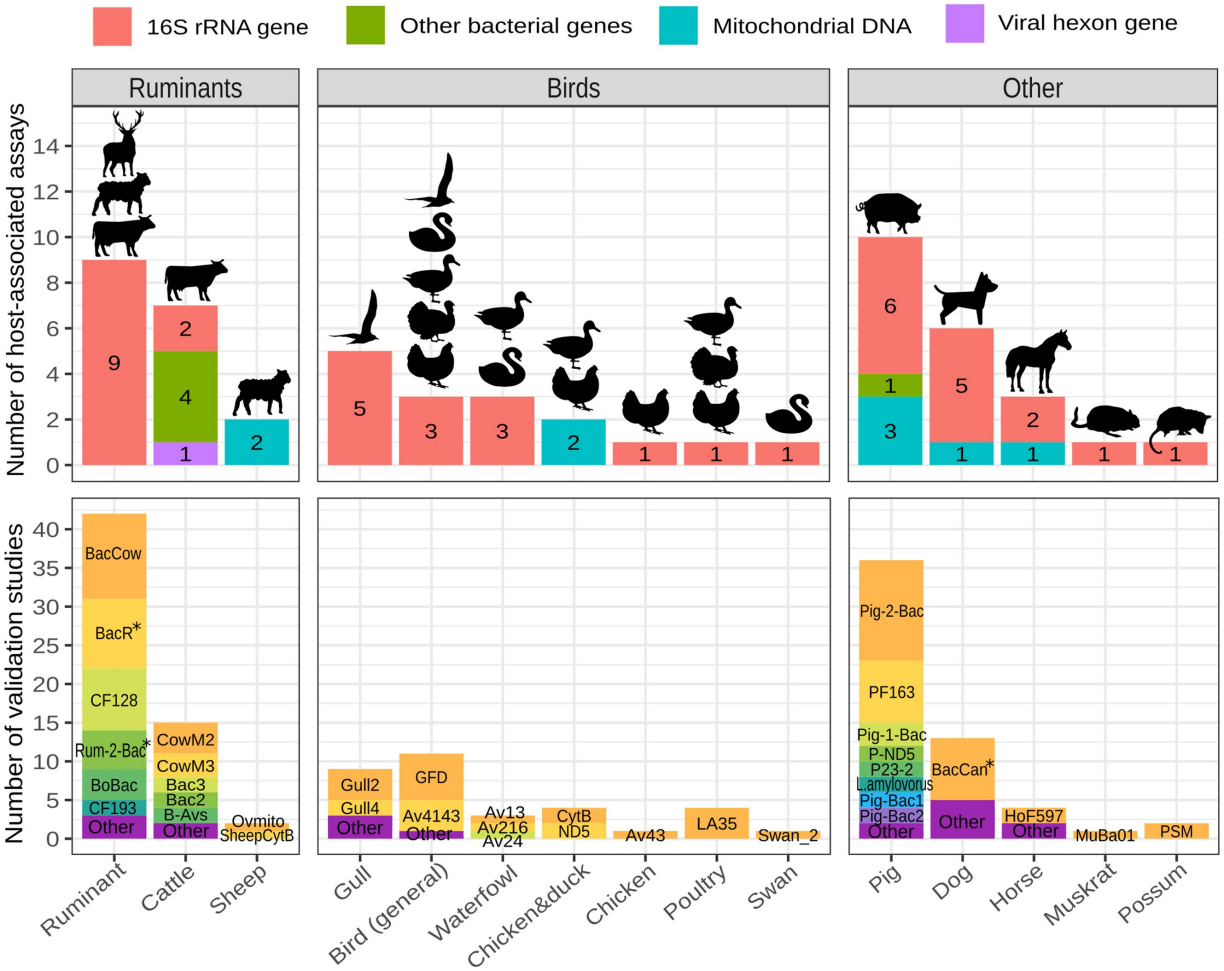
Number of host-associated assays and validation studies per animal host. Assays targeting various hosts across different studies are grouped into the host category that best reflects their specificity (BoBac and BacCow are categorized as ruminant-associated; Bac2, Bac3, CowM2 and CowM3 are categorized as cattle-associated; GFB and GFC are categorized as gull-associated and GFD is categorized bird-associated). The figure was created with R 4.2.2 using packages ggplot2, ggfittext and scales. The icons were made by Freepik and obtained from www.flaticon.com. *the validations included either original or modified assays.

The majority of published animal-associated assays are designed to detect fecal contamination originating from pigs, ruminants (domestic and/or wild), cattle, birds, and dogs. When focusing on bird-associated assays, most of them aim to identify fecal contamination originating from gulls, followed by birds in general, waterfowl, chickens, ducks, poultry, and swans. Few assays have been developed to identify fecal contamination originating from wild animals apart from birds. They target muskrats and possums (Figure 3; Supplementary Table 1). General ruminant-associated assays are typically utilized for detecting fecal contamination deriving from domestic ruminants, but they are also capable of detecting feces of wild ruminants (Fremaux et al., 2009; Mieszkin et al., 2010; Raith et al., 2013; Reischer et al., 2013; Somnark et al., 2018a; Kolm et al., 2019; Ballesté et al., 2020; Zhang et al., 2020).

The animal-associated assays described above were validated across 46 different studies, collectively resulting in 170 validations. General ruminant and pig-associated assays are by far the most validated, followed by assays targeting cattle, dogs, birds in general, gulls and waterfowl (Figure 3; Supplementary Table 2). The most frequently validated assays with general information are shown in Table 1.

**Table 1:** Most frequently validated assays of animal fecal pollution.

Target host	DNA target	Target organism	Assay	Sensitivity	Specificity	Original referencesˣ	Validation references
Ruminants (general)	16 s rRNA gene	*Bacteroidales*	**BacCow**	63 – 100	0 – 91	Bernhard and Field (2000a), Kildare et al. (2007)	Reischer et al. (2006), Kildare et al. (2007), Ahmed et al. (2010), Raith et al. (2013), Odagiri et al. (2015), Symonds et al. (2017), Haramoto and Osada (2018), Malla et al. (2018), Somnark et al. (2018a), Vadde et al. (2019), Zhang et al. (2020)
			**BacR**	91 – 100	84 – 100	Reischer et al. (2006), Kolm et al. (2019)	Reischer et al. (2006), Mieszkin et al. (2009), Marti et al. (2011), Raith et al. (2013), Haramoto and Osada (2018), Malla et al. (2018), Kolm et al. (2019), Linke et al. (2021)
			**CF128**	85 – 100	0 – 96	Bernhard and Field (2000a, 2000b)	Gawler et al. (2007), Dorai-Raj et al. (2009), Fremaux et al. (2009), Shanks et al. (2010), Kirs et al. (2011), Somnark et al. (2018a), Ballesté et al. (2020), Zhang et al. (2020)
			Rum-2-Bac*	94 – 100	69 – 100	Mieszkin et al. (2009), Yahya et al. (2017)	Mieszkin et al. (2010), Raith et al. (2013), Yahya et al. (2017), Zhang et al. (2020), Rytkönen et al. (2021)
			**BoBac**	82 – 100	5 – 100	Layton et al. (2006)	Layton et al. (2006), Shanks et al. (2010), Reischer et al. (2013), Somnark et al. (2018a)
Cattle	Gene encoding a HDIG domain protein	*Bacteroidales*	**CowM2**	50 – 100	0 – 100	Shanks et al. (2008)	Shanks et al. (2010), Raith et al. (2013), Odagiri et al. (2015), Somnark et al. (2018a)
Pigs	16S rRNA gene	*Bacteroidales*	**PF163**	87 – 100	9 – 100	Bernhard and Field (2000b), Dick et al. (2005a)	Fremaux et al. (2009), Lamendella et al. (2009), Symonds et al. (2017), Haramoto and Osada (2018), Malla et al. (2018), Somnark et al. (2018a), Ballesté et al. (2020), Xu et al. (2020)
			**Pig-1-Bac**	98 – 100	68 – 100	Mieszkin et al. (2009)	Mieszkin et al. (2009), Xu et al. (2020), Zhang et al. (2020)
			**Pig-2-Bac**	75 – 100	66 – 100	Mieszkin et al. (2009)	Mieszkin et al. (2009), Marti et al. (2011), He et al. (2016), Haramoto and Osada (2018), Malla et al. (2018), Somnark et al. (2018b), Vadde et al. (2019), Ballesté et al. (2020), Schiaffino et al. (2020), Xu et al. (2020), Zhang et al. (2020), Linke et al. (2021), Rytkönen et al. (2021)
Birds (general)	16 s rRNA gene	*Helicobacter*	**GFD**	44 – 88	56 – 100	Green et al. (2012)	Green et al. (2012), Ahmed et al. (2016a), Symonds et al. (2017), Vadde et al. (2019), Zhang et al. (2020), Rytkönen et al. (2021)
		*Lactobacillus*	**Av4143**	57 – 100	82 – 97	Ohad et al. (2016)	Ohad et al. (2016), Vadde et al. (2019), Schiaffino et al. (2020), Rytkönen et al. (2021)
Poultry	16S rRNA gene	*Brevibacterium avium*	**LA35**	23 – 78	91 – 100	Weidhaas et al. (2010), Weidhaas and Lipscomb (2013)	Weidhaas et al. (2010), Weidhaas and Lipscomb (2013), Schiaffino et al. (2020)
Gulls	16S rRNA gene	*Catellicoccus marimammalium*	**Gull2**	13 – 100	86 – 100	Lu et al. (2008)	Kirs et al. (2011), Ryu et al. (2012), Symonds et al. (2017), Ballesté et al. (2020)
Dogs	16S rRNA gene	*Bacteroidales*	BacCan*	76 – 100	45 – 100	Kildare et al. (2007)	Kildare et al. (2007), Odagiri et al. (2015), Nshimyimana et al. (2017), Symonds et al. (2017), Malla et al. (2018), Schiaffino et al. (2020), Rytkönen et al. (2021), Yasar et al. (2021)

Only assays validated in three or more publications are listed in this table. Additional information about all assay validations can be found in Supplementary Table 2. This includes the information about target and non-target hosts tested, primer and probe names and sequences, amplicon lengths, molecular techniques used, PCR conditions, other validation metrics, technical and analytical measures, biological measures, and sampling locations.

*forward and/or reverse primer were modified in one or more validation studies; ˣreferences for original and modified assay development.

Methodological differences have been observed among validation studies including the utilization of various amplification-based technologies (end-point PCR, qPCR, HDA), detection chemistry (SYBR green dye, TaqMan probes), probes and annealing temperatures. Most papers employed qPCR and end-point PCR for assay validation, with none of the studies incorporating LAMP technology, despite its increasing prominence (Supplementary Table 2).

### 6.1 Ruminant-associated fecal source tracking assays

As the meat and dairy industry expands, more domesticated ruminants such as cattle, sheep, and goats are being raised in factory farms. These concentrated populations can act as point sources of fecal pollution if proper management practices are not in place. However, it is also important to note that some domestic ruminants graze on grass outside of farms, particularly in extensive grazing systems or open rangelands. In such cases, their waste may be deposited directly onto the land, potentially entering water bodies through runoff during rainfall events (Garcia-Armisen and Servais, 2007). In addition to domestic ruminants, the contribution of wild ruminants to fecal contamination of surrounding waters should not be underestimated (Nguyen et al., 2018).

Among ruminant-associated FST assays some target ruminant feces in general (CF128, BacR, Rum-2-Bac, BacCow, BoBac) while others aim to target cattle (CowM2, CowM3, B-Avs) or sheep feces (Ovmito, SheepCytB) (Figure 3; Supplementary Table 1). BacCow was originally classified as a cattle-associated marker (Kildare et al., 2007) but is now generally considered ruminant-associated (Raith et al., 2013). Another marker, BoBac was published as bovine-associated (Layton et al., 2006) and is now considered to be associated with ruminants in general (Reischer et al., 2013).

All assays associated with ruminants in general target the 16S rRNA gene of *Bacteroidales* (Figure 3; Supplementary Table 1). Among them BacCow, BacR, CF128 and Rum-2-Bac are most frequently employed and validated (Figure 3). BacR has consistently shown very high performance in terms of sensitivity (>90%) and specificity (>84%) (Reischer et al., 2006; Mieszkin et al., 2009; Marti et al., 2011; Raith et al., 2013; Haramoto and Osada, 2018; Malla et al., 2018; Kolm et al., 2019; Linke et al., 2021). Rum-2-Bac was also highly specific (>96%) and sensitive (>93%) in multiple studies (Mieszkin et al., 2010; Raith et al., 2013; Yahya et al., 2017; Rytkönen et al., 2021), except when tested in China, where its sensitivity was 69% (Zhang et al., 2020). Numerous studies have consistently reported low specificity of the BacCow marker even when considered ruminant-associated (Reischer et al., 2006; Kildare et al., 2007; Ahmed et al., 2010; Raith et al., 2013; Odagiri et al., 2015; Symonds et al., 2017; Haramoto and Osada, 2018; Malla et al., 2018; Somnark et al., 2018a; Vadde et al., 2019; Zhang et al., 2020). Odagiri et al. (2015) even broadened its target host range to include domestic animals and ruminants, raising doubts about its overall utility. The assay did however discriminate between human and livestock/domestic animal feces. Similarly, although highly sensitive, the CF128 assay often resulted in low specificity (Gawler et al., 2007; Fremaux et al., 2009; Shanks et al., 2010; Kirs et al., 2011; Somnark et al., 2018a; Ballesté et al., 2020; Zhang et al., 2020). Other assays associated with ruminant feces include BoBac, CF193, Bac32F/RumD1Rm, Bac32F/RumD2R and RumB1F/BacPreR. Among them, all except BoBac and CF193 have been found to be highly accurate in terms of both, specificity and sensitivity, however they have only been validated once (Dorai-Raj et al., 2009; Shanks et al., 2010; Reischer et al., 2013; Somnark et al., 2018a). General ruminant assays are known to detect feces of wild ruminants including deer, caribou, chamois, ibex, moose, and bison. Furthermore, they may detect some closely related non-ruminants and pseudoruminants such as llamas and camels (Fremaux et al., 2009; Mieszkin et al., 2010; Raith et al., 2013; Reischer et al., 2013; Somnark et al., 2018a; Kolm et al., 2019; Ballesté et al., 2020; Zhang et al., 2020).

Seven FST assays have been designed to detect and trace fecal pollution originating from cattle. Two target the 16S rRNA gene of *Bacteroidales* (Cow-Bac2 EP) and *Bifidobacterium* (CWBif), four target other bacterial genes (Bac2, Bac3, CowM2, CowM3) and one (B-Avs) targets a gene encoding the Hexon protein of an adenovirus. The B-Avs assay was published as bovine associated (de Motes et al., 2004), however, validation results are not yet published on bovines other than cattle. Based on previous results, Bac2, Bac3 and B-Avs were shown to be highly specific, but variable results were obtained on sensitivity (Ahmed et al., 2010, 2013; Shanks et al., 2010; Somnark et al., 2018a). Results on performance of CowM2 are contradictory (Riedel et al., 2014; Odagiri et al., 2015; Somnark et al., 2018a), while CowM3 showed high specificity and sensitivity in two separate studies (Ahmed et al., 2013; Raith et al., 2013). Cow-Bac2 and CWBif were validated once with results pointing to low specificity (Yahya et al., 2017; Somnark et al., 2018a).

Two sheep associated FST assays target host mtDNA (Ovmito, SheepCytB). Both were validated only once. They showed high specificity and sensitivity and are therefore potential markers for detecting fecal contamination originating from sheep (Ballesté et al., 2020; Rytkönen et al., 2021). Caution should be taken when interpreting results of mtDNA based assays as mtDNA can originate from all eukaryotic cells, possibly resulting in false positive results.

Apart from domesticated ruminants, wild ruminants should also be considered for FST purposes, depending on the geographic location. Native to all continents except Antarctica and Australia (Hernández Fernández and Vrba, 2005), wild ruminants represent a crucial part of ecosystems and often live in herds, increasing the potential for contaminating waterways in their proximity. Feral ruminants have also established wild populations in Australia following their introduction during European colonization (Skeat et al., 1996; Forsyth et al., 2019).

### 6.2 Pig-associated fecal source tracking assays

As an important agricultural subsector, pig farming produces a substantial amount of waste. If not properly managed, it can spread to the environment and expose us to a variety of zoonotic pathogens, most notably *E. coli*, *Salmonella*, *Campylobacter*, *Yersinia*, *Cryptosporidium*, and *Giardia* (Guan and Holley, 2003; Massé et al., 2011). Using pig-associated FST markers we can identify contaminated areas and address any improper management of pig waste (Heaney et al., 2015).

Among ten pig-associated assays found, six target the 16S rRNA gene (Pig-2-Bac, Pig-1-Bac, PF163, *L.amylovorus*, Pig-Bac1, Pig-Bac2), three target pig mtDNA (Pomito, P-ND5, P-CytB) and assay P23-2 targets a methyl-coenzyme M reductase gene in methanogenic bacteria (Figure 3; Supplementary Table 1). Pig-2-Bac was the most validated (Figure 3; Supplementary Table 2) and proved superior to other assays such as Pig-Bac1, Pig-Bac2, *L. amylovorus*, P-CytB and P-ND5 on multiple occasions in terms of sensitivity and specificity (Mieszkin et al., 2009; He et al., 2016; Xu et al., 2020; Zhang et al., 2020). Contradictory results on performance were obtained when comparing Pig-2-Bac to the PF163 assay (Haramoto and Osada, 2018; Ballesté et al., 2020; Xu et al., 2020). Xu et al. (2020) tested five pig-associated assays in China and Mongolia and found Pig-2-Bac, Pig-1-Bac and PF163 to have equally high performance (100% sensitivity and specificity) while Pig-Bac1 and Pig-Bac2 assays resulted in exceptionally low specificity. Other assays requiring more validation studies include P23-2 (Lamendella et al., 2007; Ufnar et al., 2007), P-ND5 (Caldwell et al., 2007; He et al., 2016; Zhang et al., 2020) and Pomito (Martellini et al., 2005; Ballesté et al., 2020).

### 6.3 Bird-associated fecal source tracking assays

Birds are known to carry human pathogens that are excreted with fecal waste and include enteric bacteria (*Salmonella*, *E. coli*, and *Campylobacter*), protozoans (*Cryptosporidium*, *Giardia*) and microsporidia (*Enterocytozoon*, *Encephalitozoon*) (Vlahović et al., 2004; Graczyk et al., 2008). There are two main potential origins of bird fecal pollution: poultry farms and wild birds. While wild bird droppings appear to harbor less abundant and fewer pathogenic bacteria than poultry, their contribution to fecal contamination of water should not be neglected as they are fundamental components of the aquatic ecosystem (Benskin et al., 2009; Boukerb et al., 2021).

We found five assays for tracking fecal contamination originating from birds in general. All of them target the 16S rRNA gene of different bacteria (Table 1; Figure 3; Supplementary Table 1). Given that birds are the most diverse land vertebrates and can be endemic to certain geographic locations (Chiappe, 2009), the selection of broadly specific markers and detecting bird feces in general can be difficult. The primary challenge lies in the sensitivity of assays, which may further decrease by validating on a broader range of wild bird species. GFD and Av4143 were the most frequently validated assays for identifying fecal contamination originating from birds (Figure 3) and showed the highest performance when compared to the remaining three markers (GFB, GHC, Av163F) (Green et al., 2012; Ohad et al., 2016). However, their performance varied greatly among different validation studies (Ohad et al., 2016; Symonds et al., 2017; Vadde et al., 2019; Schiaffino et al., 2020; Zhang et al., 2020; Rytkönen et al., 2021) and within-study comparisons show contradictory results on which assay results in highest specificity and sensitivity (Supplementary Table 2; Vadde et al., 2019; Rytkönen et al., 2021).

Some assays were designed to detect gull feces. Among them Gull2 and Gull4 were the most frequently validated (Figure 3). According to the results of Ryu et al. (2012), Gull4 assay tends to be more specific and less sensitive than Gull2. GHC and GFB assays for the detection of gull feces were validated only when first published by Green et al. (2012). The specificity of these assays was very high, however they detected only 64 and 26% of gull feces, respectively.

For detecting fecal contamination of waterfowl, Ohad et al. (2016) developed three assays with relatively low sensitivity but high specificity: Av13, Av24 and Av216. Using the comparative analysis of the 16S rRNA gene, one swan-associated (Swan_2) marker was developed and resulted in sensitivity of 75% and specificity of 90% when tested on fecal samples (Boukerb et al., 2021).

Among assays developed for the detection of fecal contamination originating from domestic birds, one assay aims to detect poultry feces in general (LA35) (Weidhaas et al., 2010; Weidhaas and Lipscomb, 2013; Schiaffino et al., 2020). Another more recently developed assay (Av43) detects chicken feces with a high degree of specificity (Ohad et al., 2016), whereas two assays based on mtDNA (ND5, CytB) aim to detect both chicken and duck feces (Zhuang et al., 2017; Schiaffino et al., 2020).

### 6.4 Fecal source tracking assays targeting other animals

While livestock farms present major pollution risk due to the large number and density of farm animals, pets such as dogs and cats should not be neglected as they are an important part of urban environments. Dog feces were previously reported to be important sources of fecal contamination in surface waters (Ervin et al., 2014; McKee et al., 2020). BacCan marker is the most validated dog-associated FST marker with varying results on performance. Sensitivity of this marker was always above 75% while specificity ranged from 47 to 100% in different studies (Kildare et al., 2007; Odagiri et al., 2015; Nshimyimana et al., 2017; Symonds et al., 2017; Malla et al., 2018; Schiaffino et al., 2020; Rytkönen et al., 2021; Yasar et al., 2021). This assay originally includes two reverse primers (Kildare et al., 2007), however in some studies only one was included (Rytkönen et al., 2021; Yasar et al., 2021). Alternatives to the BacCan assay include DogND5 targeting dog mtDNA (Caldwell and Levine, 2009; Rytkönen et al., 2021) and DogBac (Dick et al., 2005b; Symonds et al., 2017), DF113F-DF472R, DF53F-DF606R and DF53F-DF606R (Hussein et al., 2014) targeting the 16S rRNA gene of *Bacteroidales* (Figure 3).

Horses, either wild or domesticated are another potential source of fecal contamination. Three horse-associated FST assays were found but lack validation studies. They target either the 16S rRNA gene of *Bacteroidales* (HoF597, HorseBact) (Dick et al., 2005a; Symonds et al., 2017; Ballesté et al., 2020) or horse mtDNA (HorseCytB) (Schill and Mathes, 2008; Rytkönen et al., 2021).

In the process of identifying new markers of fecal pollution, there is often a tendency to overlook wild animals. Only two assays have been developed with the aim to detect feces of wild animals other than birds and they target muskrats and possums (Marti et al., 2011; Devane et al., 2013). Given that muskrats live in riparian areas and excrete in the water, they are particularly relevant in terms of direct contamination of water sources. They can spread certain pathogens including *G. duodenalis* and *Cryptosporidium* spp. (Erlandsen et al., 1990; Marti et al., 2011). Marti et al. (2011) developed an assay, designated MuBa01 for identifying fecal pollution deriving from muskrats. It was detected in 66% of muskrat samples and did not cross-react with samples of other hosts. Another assay was developed to target feces of possums, one of New Zealand’s most serious mammalian pests with initial validation resulting in 83% sensitivity and 96% specificity (Devane et al., 2013).

## 7 Conclusion

The most common way to determine sources of fecal pollution involves detecting host-associated markers found within bacterial, viral, or mitochondrial nucleic acids. In this review we presented 55 assays designed for the detection of non-human animal fecal pollution alongside validation results from 41 studies. Based on gathered information we found that several promising markers for non-human FST have already been discovered, however there is still a potential for further exploration, especially for determining wildlife sources of fecal pollution. Regarding existing markers, more in depth knowledge is required to understand their key biological attributes, including persistence in the environment (considering biotic and abiotic factors), correlation to human pathogens, resistance to technical and chemical treatment and movement patterns. To improve evaluation of FST marker diagnostics, it is important to expand reference collections and include samples from a wider range of geographic locations. Furthermore, we found lack of standardization of protocols and inconsistencies in data reporting, making validation results difficult to compare. The most significant disparities in methodology and data reporting were observed in establishing sLOD and sLOQ.

A limited number of validation studies investigated the potential influence of the environmental matrix on assay performance by assessing sLOD and sLOQ. Among these studies, discrepancies were observed regarding the type of matrix taken into consideration - whether it was solely fecal or a combination of fecal and water. Because the aim of FST validations is to later identify markers in environmental water, we think that the water matrix should be accounted for during assay validation. Therefore, we suggest reporting sLOD and sLOQ in units of fecal weight per volume of environmental water of interest to enhance consistency across studies. These parameters can be evaluated in different water sources (e.g., river, lake, groundwater, seawater).

Differences in assay sensitivity and specificity were noticeable across various studies, and they can be attributed to a multitude of factors. They can arise from variations in the methodologies applied, variations in sampling intensity, the selection of host species for specificity determination and the choice of detection limits. Additionally, geographical location and time of sampling can play a significant role, as some markers are known to exhibit temporal and geographical instability. One effective strategy to increase diagnostic accuracy measures is to detect multiple markers associated with a particular host simultaneously.

Ultimately, it is important to acknowledge that FST does not offer a universally applicable solution. Instead, it operates as a toolbox approach that is required to navigate a diverse array of markers and methodology according to each individual situation. The information gathered in this review can serve as a starting point for choosing appropriate assays for determining non-human sources of fecal pollution.

## Author contributions

TZ: Writing – original draft, Data curation, Visualization. MR: Writing – review & editing, Conceptualization, Supervision, Funding acquisition.
